# Hormone Receptor-Positive/HER2-Positive Breast Cancer: Hormone Therapy and Anti-HER2 Treatment: An Update on Treatment Strategies

**DOI:** 10.3390/jcm13071873

**Published:** 2024-03-24

**Authors:** Chiara Tommasi, Giulia Airò, Fabiana Pratticò, Irene Testi, Matilde Corianò, Benedetta Pellegrino, Nerina Denaro, Laura Demurtas, Mariele Dessì, Sara Murgia, Giovanni Mura, Demi Wekking, Mario Scartozzi, Antonino Musolino, Cinzia Solinas

**Affiliations:** 1 Medical Oncology and Breast Unit, University Hospital of Parma, 43126 Parma, Italy; giulia.airo@unipr.it (G.A.); fabiana.prattico@unipr.it (F.P.); irene.testi@unipr.it (I.T.); matilde.coriano@unipr.it (M.C.); benedetta.pellegrino@unipr.it (B.P.); 2 Department of Medicine and Surgery, University of Parma, 43121 Parma, Italy; 3 GOIRC (Gruppo Oncologico Italiano di Ricerca Clinica), 43100 Parma, Italy; 4 Medical Oncology, Fondazione IRCCS Ca’ Granda Ospedale Maggiore Policlinico, 20122 Milano, Italy; 5 Medical Oncology, AOU Cagliari, Policlinico Duilio Casula, 09042 Monserrato, Italyczsolinas@gmail.com (C.S.); 6 Medical Oncology, University of Cagliari, 09124 Cagliari, Italy; 7 Pathological Anatomy, Laboratory Valdès, 81200 Cagliari, Italy; 8 Academic Medical Centre, Amsterdam University Medical Center, University of Amsterdam, 1098 XH Amsterdam, The Netherlands

**Keywords:** hormone receptor positive HER2-positive breast cancer, endocrine therapy, estrogen receptor, HER2, HER2 blockage

## Abstract

Hormone receptor (HR)-positive/HER2-positive breast cancer represents a distinct subtype expressing estrogen and progesterone receptors with an overexpression of HER2. Approximately 14% of female breast cancer cases are HER2-positive, with the majority being HR-positive. These tumors show a cross-talk between the hormonal and HER2 pathways; the interaction has implications for the treatment options for the disease. In this review, we analyze the biology of HR-positive/HER2-positive breast cancer and summarize the evidence concerning the standard of care options both in neoadjuvant/adjuvant settings and in advanced disease. Additionally, we focus on new trials and drugs for HR-positive/HER2-positive breast cancer and the new entity: HER2-low breast cancer.

## 1. Introduction

Cells of hormone receptor (HR)-positive/human epidermal growth factor receptor 2 (HER2)-positive breast cancer (BC) not only express estrogen receptors (ER) in conjunction with or without progesterone receptors (PR) but may also overexpress HER2 on their cell surface or exhibit an amplification of its gene [1]. HER2-positive BCs constitute about 14% of all female BC cases, with 18.8 new diagnoses per 100,000 women; among these, the majority are HR-positive (HR-positive = 13.3 versus HR-negative = 5.5), and the 5-years survival is better than that of patients with HR-negative/HER2-positive BC in all settings of the disease [2].

The presence of a cross-talk between HER2 signaling pathways and ER requires treatments that target the different pathways to achieve synergic effects in terms of response. Different therapeutic options are available for HR-positive/HER2-positive BC in early settings as well as in advanced disease, and different ongoing trials aim to understand the best approach for these patients.

In addition, recent evidence about new drugs show that immunohistochemistry (IHC) 1+ or 2+ with negative in situ hybridization (ISH) HER2 BC, namely, HER2-low BC, can benefit from HER2-targeted therapy. In particular, the antibody-drug conjugated (ADC) trastuzumab deruxtecan (T-DXd) [3] is expanding the armamentarium against BC. This development contributes to improving BC knowledge and therapeutic options.

## 2. Biology and Prognosis of Hormone Receptor-Positive/HER2-Positive Breast Cancer

The crosstalk between the ER and HER2 pathways not only plays a role in resistance to endocrine agents but also in resistance to HER2-directed agents. Various pre-clinical and clinical data demonstrate that HER2-positive BC that express HR exhibit particular biology with intrinsic resistance to endocrine therapy (ET) [4].

**Figure 1:**
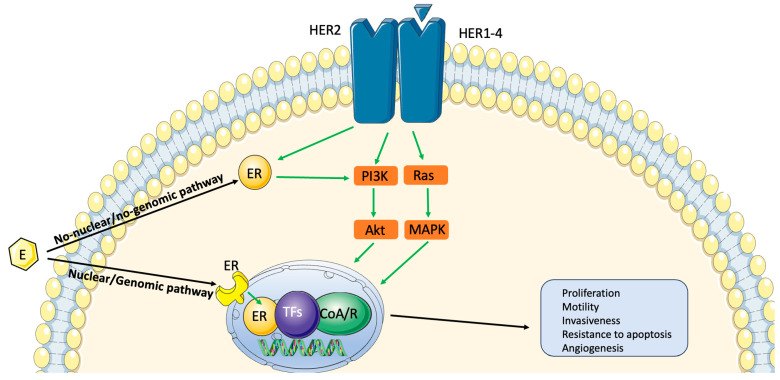
Convergence of HER2 and ER pathways in HR-positive/HER2-positive BC. Green arrows show the stimulated pathways after HER2 and HR stimulation (explanation in the text).

ER signaling is the dominant driver of cell proliferation and survival. Ligand-dependent ER activation triggers a cascade that brings to the stimulation of (1) the non-nuclear/non-genomic pathway with the promotion of kinases phosphorylation, in particular phosphatidylinositol 3-kinase (PI3K); (2) the nuclear/genomic pathways, in which the ligand–receptor complex binds specific DNA regions and induces the transcription of ER-related genes and the proliferation of cells [5]. The genomic pathway is predominant in tumor cells and serves as the target of ET. Non-genomic ER activity increases and amplifies the HER2 pathway [6]. HER2 activation through heterodimerization induces the phosphorylation of ERs and cytoplasmic tyrosine kinases, leading to the activation of their downstream pathways such as mitogen-activated protein kinase (MAPK), protein kinase B (AKT), and PI3K [7]. In the presence of hyperactive HER signaling, activated downstream kinases reduce ER expression and, simultaneously, phosphorylate ER, reducing the effect of ET [6] (Figure 1).

Chen et al. evaluated the impact of ER level and its impact on prognosis in HR-positive/HER2-positive BC; they distinguished ER expression as low (1–9%), median (10–79%), and high (80–100%). In the analyses, only a high ER level was strongly correlated with a better survival outcome (hazard ratio 0.09, 95%CI 0.01–0.68, *p* = 0.019) and the benefit continues to remain meaningful after adjustment for tumoral dimension and nodal status [8].

The complexity of heterogeneity of HR-positive/HER2-positive BC impacted the outcome of patients; in fact, HR-positive/HER2-positive BC had significantly better BC-specific survival (all log-rank *p* < 0.001) despite HR-negative/HER2-positive BC. They showed a significantly lower TP53 mutation rate (30% vs. 69% in HR-negative/HER2-positive BC, *p* < 0.001) and an increased rate of MUC16, GATA3, and ERBB3 mutations. HR-positive/HER2-positive BC showed significantly lower rates of ERBB2 and MYC amplification, HER2 protein, and phosphorylated HER2 protein levels [9]. The gene expression profile analyses showed the luminal B subtype was the most common, followed by the HER2-enriched and luminal A subtypes [10].

Pre-clinical evidence shows that the combination of anti-HER2 and anti-ER therapies enhances the response to the treatments and delays the insurgence of endocrine resistance [4]. However, the clinical benefit is modest, suggesting that alternative survival pathways in BC cells bypass the bidirectional cross-talk between HER2 and ER [11].

In the first 5 years after diagnosis, early HR-negative/HER2-positive patients have a worse survival rate compared with the triple-positive group (85.6% vs. 91%, respectively) [2]. Furthermore, the 5 years-recurrence risk is similar between the two groups, particularly in patients with no or minimum nodal involvement [12]. Other evidence suggested that high ER and PR expression levels impact on response to chemo- and trastuzumab-based adjuvant treatments; in addition, patients with ER > 50% had a worst prognosis, despite patients with ER < 50% (5-year DFS was 75.4% and 80.8% and 7-year DFS was 67.1% and 78%, respectively, *p* < 0.001) [13].

A new literature review tried to identify factors associated with recurrence in patients with HER2-positive early BC. No pathologic complete response (pCR) after neoadjuvant chemotherapy (CT) and fewer basal tumor-infiltrating lymphocytes (TILs) are the most represented risk factors of recurrence. HR status remained an important risk factor for recurrence, with HR-positive/HER2-positive disease more likely to recur. Nevertheless, the association between HR status and HER2-positive BC recurrence is highly context dependent (e.g., adjuvant/neoadjuvant treatment and use of endocrine therapy combined with other treatment) [14].

## 3. Early Stages

### 3.1. Neoadjuvant Setting

Preoperative systemic therapy represents the standard approach in the treatment of locally advanced and operable BC greater than 2 cm (>cT2) and/or with nodal involvement [15]. It is as effective as adjuvant CT in terms of disease-free (DFS) and overall survival (OS), and it increases the rate of breast-conserving surgery [16,17]. Patients who exhibit an objective tumor response to primary CT, particularly those with a pCR, have improved long-term cancer outcomes compared to patients who do not respond or have residual invasive tumors [18]. TILs have emerged as important prognostic/predictive factors, especially in HER2-positive BC [19]. A meta-analysis of randomized controlled trials demonstrated that patients with high TILs had a significant increase in pCR rate regardless of the antiHER2 agent used in the neoadjuvant setting [20].

Neoadjuvant trastuzumab should be given to all HER2-positive patients, with the possible exception of selected very-low-risk cases, such as T1a N0 tumors. It is administered with CT, resulting in high rates of pCR, as demonstrated in the NOAH trial (pCR rates: 38% in the trastuzumab group vs. 19% in the control group) [21]. Nevertheless, patients with negative HRs demonstrated a more pronounced benefit in terms of pCR from trastuzumab, with an association between pCR and both DFS and OS [22].

Double blockade with trastuzumab and pertuzumab in high-risk patients was evaluated in the NeoSphere trial. Patients treated with pertuzumab and trastuzumab plus docetaxel had a significantly improved pCR rate of 45.8%. In this context, patients with HR-negative BC had a higher pCR rate compared to those patients with HR-positive BC (63.2% vs. 26.0%, respectively) [23].

The benefit of dual HER2-targeting has also been observed in the NeoALTTO trial, where patients treated with neoadjuvant trastuzumab and tyrosine kinase inhibitor (TKI) lapatinib plus CT had a pCR rate of 51.3%, compared with 29.5% of patients treated with trastuzumab plus CT. Data confirmed that the HR-positive subgroup had a lower pCR rate compared to the HR-negative one [24].

The ADAPT HER2-positive/HR-positive trial tested trastuzumab emtansine (T-DM1), with or without ET, for 12 weeks in the neoadjuvant setting: the treatment with an ADC result in a clinically meaningful pCR rate (41% treated with T-DM1 versus 15.1% treated with trastuzumab plus ET). The ET-ADC combination does not seem to play a crucial role in pCR [25].

Nevertheless, antiHER2 plus CT combination are recommended for all cases of HER2-positive early-stage BC regardless of ER status. In any case, the benefit of neoadjuvant approach in stage I (T1a/bN0) HER2-positve BC in unclear. In the last years, the number of patients with stage I HER2-positive BC receiving neoadjuvant treatment has increased, particularly in the case of cT1c tumors. Among factors evaluated in therapeutic decision processes, HR status seems to play a crucial role: the neoadjuvant approach, in fact, is preferred in HR-negative BC [26].

Because of the low efficacy of CT approaches in HR-positive/HER2-positive BC, new chemo-free strategies are emerging; the NA-PHER2 trial evaluated the neoadjuvant use of palbociclib, fulvestrant, trastuzumab, and pertuzumab in patients with early HR-positive/HER2-positive BC with clinical objective response in 97% and 27% of pCR in enrolled patients [27]. Other clinical trials are evaluating the role of chemo-free approaches in early HER2-positive BC (e.g., TOUCH [28], PHERGAIN-2 [29], and NCT05228951 trials [30]).

### 3.2. Adjuvant Setting

Two trials, led by the National Surgical Adjuvant Breast and Bowel Project (NSABP) and the North Central Cancer Treatment Group (NCCTG), demonstrated a clear benefit of trastuzumab added to anthracycline and taxane-based CT in the adjuvant setting, with significantly improved DFS rates [31]. The benefit leads to a significant and long-lasting improvement in survival, reducing the risk of cancer recurrence, and the advantage was observed across all subgroups of patients (hazard ratio 0.51, 95% CI 0.39–0.67, in HR-positive patients) [32].

Several trials have also focused on determining the optimal duration of trastuzumab treatment. The HERA trial demonstrated that 2 years of trastuzumab were not more effective than 1 year of trastuzumab, supporting the use of 1 year of treatment as the standard of care [33]. HR status remains a significant prognostic factor: women with HR-negative disease experience a higher rate of recurrences and deaths, even after a median follow-up of 11 years. However, there is no evidence that the efficacy of anti-HER2 therapy is different according to HR status [34].

Greater anti-tumor activity is observed when combining two anti-HER2 agents. The APHINITY trial demonstrated that the addition of pertuzumab to adjuvant trastuzumab and CT significantly improved invasive disease-free survival (IDFS) for patients with node-positive HER2-positive BC (hazard ratio 0.76, 95% CI 0.64–0.91), regardless of HR status (hazard ratio 0.73, 95% CI 0.59–0.92 in HR-positive group) [35]. Conversely, the ALTTO trial did not demonstrate the benefit of adjuvant lapatinib, with or without trastuzumab, regardless of the hormone receptor subgroup [36].

Extended anti-HER2 therapy with neratinib, an irreversible pan-HER tyrosine kinase inhibitor, may be considered in selected high-risk patients based on the findings from the ExteNET trial. Herein, neratinib, administered for 1 year after trastuzumab-based therapy, significantly improved IDFS in women with high-risk early-stage HER2-positive BC [37]. Subgroup analysis showed that neratinib provided greater benefit to patients with HR-positive BC (hazard ratio 0.51, 95% CI 0.33–0.77, *p* = 0.0013) compared to those with HR-negative disease (hazard ratio 0.93, 95% CI 0.60–1.43, *p* = 0.74; *p* interaction = 0.054) [38].

In T1N0 HER2-positive BCs, the role of adjuvant trastuzumab is controversial. Age ≤ 40, T1c, and HR-positivity were identified as independent prognostic factors, and patients treated with CT plus trastuzumab could significantly benefit from the combination treatment despite observation or CT alone (hazard ratio 3.95 (*p* < 0.001) versus 2.70 (*p* = 0.034), respectively) [39]. In a retrospective analysis, adjuvant CT with trastuzumab was associated with improved DFS only in patients with T > 8 mm (>T1c) [40].

Concerning ET, the optimal approach remains controversial in patients with HR-positive/HER2-positive disease. In early-stage HR-positive BC, adjuvant treatment with the selective ER modulator tamoxifen for a duration of 5 years reduces the risk of recurrence and death by about one-third at 15 years [41]. Additionally, aromatase inhibitors (AIs) are even more effective for postmenopausal women, with further proportional reductions in recurrence rates of approximately 30% [42].

In premenopausal women, the addition of ovarian function suppression (OFS) to tamoxifen has been shown to significantly reduce the risk of recurrence and death; however, it is also associated with a worse tolerability profile, including a higher incidence of side effects such as menopausal symptoms and sexual dysfunction [43,44]. In premenopausal patients undergoing OFS, an AI is superior to tamoxifen, as demonstrated by the TEXT and the SOFT trials, with a significant improvement in the 8-year DFS compared to those assigned to receive tamoxifen plus ovarian suppression (86.8% vs. 82.8%). The OFS added to tamoxifen seems to have a stronger benefit among women with HER2-positive BC than those with an HER2-negative disease (hazard ratio 0.41 vs. 0.83, respectively; *p* = 0.04 for interaction) [45].

A large meta-analysis conducted by the EBCTCG, including data from four trials (ABCSG XII, SOFT, TEXT, and HOBOE) and involving 7030 women with HR-positive tumors, revealed a lower rate of BC recurrence in women treated with AIs plus OFS compared to those assigned to tamoxifen (relative risk RR 0.79, 95% CI 0.69–0.90, *p* = 0.0005). In a subgroup analysis, recurrence rates were similar between AIs and tamoxifen in women with HER2-positive tumors (16.0% vs. 13.9%), although only 723 (15.7%) of 4594 women with HR-positive status had HER2-positive disease [46]. Another meta-analysis, involving 5390 HR-positive/HER2-positive BC patients from six randomized trials (TEAM, ATAC, BIG 1–98, TEXT, SOFT, and ALTTO), revealed no significant difference between adjuvant treatment with AIs versus tamoxifen (hazard ratio 0.99, 95% CI 0.68–1.44, *p* = 0.96; I2 =72.9%, *p* = 0.005). However, the limited number of patients with HR-positive/HER2-positive BC included in the analysis and the small number of events precluded definitive conclusions [47]. The addition of gonadotropin-releasing hormone analogs (GnRHa) to ET for premenopausal patients aged ≤45 years was associated with a significantly better DFS [48].

Considering that HR-positive BCs are characterized by a risk of late recurrence spanning several years, and that more than half of BC recurrences occur more than 5 years after the diagnosis [49], continuing tamoxifen for 10 years, rather than stopping at 5 years, leads to a further reduction in BC recurrence, mortality and overall mortality, as shown in ATLAS and aTToM trials [50,51]. Extended treatment should be considered only for high-risk patients with HR-positive disease who have tolerated the treatment well thus far, considering potential side effects.

In premenopausal patients with HR-positive/HER2-positive BC, the risk of recurrence justifies the extension of tamoxifen, with or without OFS, for a duration of 10 years [52]. The role of extended ET with OFS + AI in high-risk premenopausal patients reduces recurrence rates; however, a substantial risk of late recurrence remains. Multiple trials evaluated the elongation of AI treatment beyond five years but evaluated only postmenopausal patients; for this reason, there is no direct evidence about the potential benefits of extended AI + OFS in premenopausal women [53,54].

In post-menopausal patients, standard ET adjuvant treatment involves AI for a duration of more than 5 years [52]. The extension of AI therapy is more controversial [55] and seems to reduce the DFS in patients with high-risk BC for nodal involvement [56]. However, there are no benefits in terms of efficacy with the prolongation of treatment until 10 years, and there is an increased risk of bone fracture for patients treated for more than 7 years with AI [51,53].

**Figure 2:**
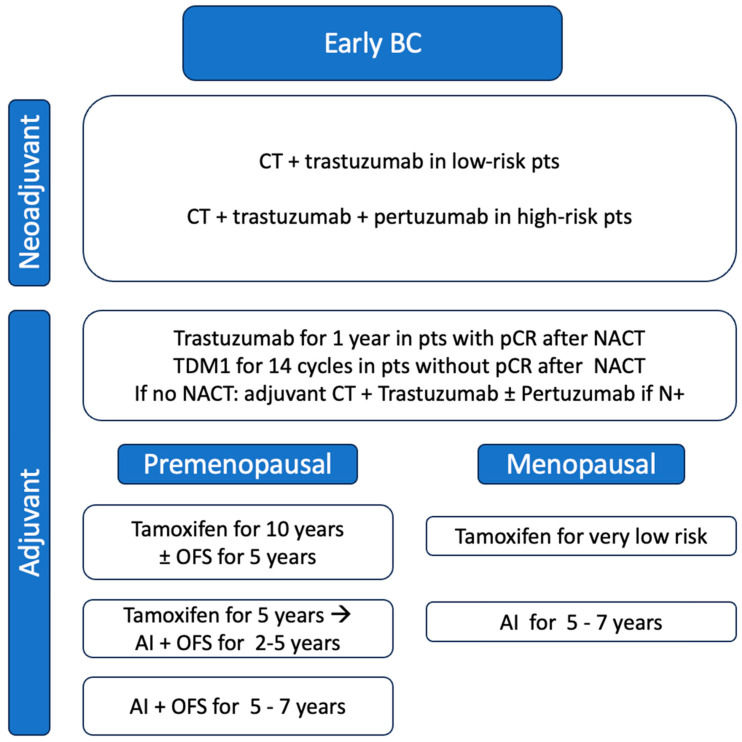
Standard treatments and endocrine therapy option in patients with HR-positive/HER2-positive early BC after antiHER2 treatments. Abbreviations: BC: breast cancer; CT: chemotherapy; pts: patients, NACT: neoadjuvant chemotherapy; pCR: pathological complete response; OFS: ovarian function suppression; AI: aromatase inhibitor.

Figure 2 summarized neoadjuvant and adjuvant treatment available in these patients.

## 4. Metastatic Setting

The use of anti-HER2 humanized monoclonal antibodies is the backbone of HER2-positive metastatic BC treatment. Currently, the standard first-line therapy is represented by the combination of pertuzumab and trastuzumab with docetaxel due to CLEOPATRA trial results; the addition of pertuzumab improves PFS (18.5 versus 12.4 months with and without pertuzumab, respectively; hazard ratio 0.62; 95% CI 0.51–0.75 months; *p* < 0.001) and OS (15.7 months longer than without pertuzumab, hazard ratio 0.69; 95% CI 0.58–0.82 months) [57]. CLEOPATRA results highlighted that patients with HR-positive/HER2-positive BC had better PFS and OS when treated with HER2-targeted therapies and CT, compared with patients with HR–negative disease [58]. Nevertheless, the treatment was administered not in combination with ET, but patients could have received one hormonal agent for metastatic BC before randomization [57]. Similar data emerged from the PERUSE trial, where ET chosen by investigators was admitted after CT in the maintenance phase. Patients with HR-positive BC showed similar PFS but longer OS despite HR-negative patients. It is worth noting that only a few of them received ET after CT discontinuation [59].

Previous studies have demonstrated that using the combination of ET to a single anti-HER2 blockade with trastuzumab (TAnDEM and eLEcTRA) or lapatinib (EGF30008) in HR-positive/HER2-positive metastatic BC led to better clinical outcomes compared with ET alone [60,61,62]. In addition, SYSUCC-002 trial showed the no-inferiority of trastuzumab and ET despite trastuzumab combined with CT as the first line of treatment in patients with HR-positive/HER2-positive metastatic BC [63]. The use of anti-HER2 TKI combined with ET did not demonstrate an advantage in this setting; the MINT trial does not show to increase endocrine responsiveness at the expense of increased skin and gastrointestinal toxicity [64].

The potential benefit of adding ET to dual anti-HER2 drugs in HR-positive/HER2-positive patients is unknown as pivotal dual anti-HER2 clinical trials precluded ET use. Pertuzumab and trastuzumab bind to different epitopes on HER2, which provides a more comprehensive signaling blockade and leads to greater activity compared with monotherapy [65]. Preclinical models have also suggested that this may inhibit HER2–ER cross-talk more efficiently, enhancing the antitumor activity of ET [66]. Clinical trials were subsequently conducted to assess the efficacy of dual anti-HER2 blockade with ET. The PERTAIN trial was the first study to explore the efficacy of adding pertuzumab to trastuzumab and an AI, along with or without induction CT, as the first line of treatment in postmenopausal patients with HR-positive/HER2-positive metastatic or locally advanced BC [67]. Dual blockade significantly improved PFS compared with trastuzumab alone. At the final analysis, the PFS benefit of pertuzumab was maintained after 6 years of follow-up, and OS was similar between arms (mPFS was 20.6 vs. 15.8 months, stratified hazard ratio = 0.67, *p* = 0.006; mOS was 60.2 vs. 57.2 months, stratified hazard ratio = 1.05, *p* = 0.78) [68].

Dual HER2 blockade plus an AI also showed better outcomes in the ALTERNATIVE clinical trial. The combination of lapatinib, trastuzumab, and an AI highlighted a PFS improvement versus trastuzumab plus an AI (mPFS was 11 vs. 5.6 months; hazard ratio = 0.62; *p* = 0.0063) in postmenopausal patients HR-positive/HER2-positive advanced BC previously treated with ET and trastuzumab plus CT in the neo(adjuvant) and/or first-line metastatic setting [69]. Even in different settings and with different agents, the PERTAIN and ALTERNATIVE results demonstrated the benefit of concurrently targeting both ER and HER2 pathways.

The introduction of cyclin-dependent kinase 4 and 6 inhibitors (CDK4/6Is) in combination with ET is the standard treatment for advanced HR-positive/HER2-negative disease [70,71,72] and preclinical data suggest that CDK4/6I are active also in HER2-positive cell lines [73,74]. In fact, clinical trials evaluated or are evaluating the benefit of adding a CDK4/6I to anti-HER2 drugs (Table 1).

The PATRICIA phase II trial aimed to evaluate the efficacy of palbociclib plus trastuzumab with or without letrozole, depending on HR status, in postmenopausal pre-treated patients with HER2-positive metastatic BC. Patients with luminal disease had better PFS than patients with non-luminal tumors, but no statistically significant differences in 6-months PFS (PFS6) were observed among the HR-positive cohorts (with and without letrozole). The PFS6 rate in HR-negative, HR-positive, and HR-positive treated-with-letrozole cohorts was 33.3%, 46.4%, and 42.8%, respectively. Regardless of the PAM50 Luminal intrinsic subtype, PFS remained better in patients with HR-positive BC: 10.6 months (95% CI, 4.1–14.8) in Luminal B, 8.2 months (95% CI, 2.2–24.1) in Luminal A, 3.8 months (95% CI, 2.1–10.9) in HER2-enriched, and 6.0 months (95% CI, 1.7–11.2) in normal-like [75].

The phase III MonarcHER trial analyzed the efficacy of abemaciclib plus trastuzumab, with or without fulvestrant, versus standard of care (CT plus trastuzumab) in HR-positive/HER2-positive advanced BC. The arm of abemaciclib plus trastuzumab plus fulvestrant showed an improved ORR and PFS compared to abemaciclib plus trastuzumab and CT plus trastuzumab arms (mPFS 8.3 vs. 5.7 vs. 5.7 months, respectively) [76]. Other trials are investigating the efficacy and safety of dual anti-HER2 blockade in association with CDK4/6I and ET. In particular, the CHEVENDO trial is a randomized phase III study comparing the safety and efficacy of trastuzumab plus pertuzumab and the CDK4/6i ribociclib in combination with either ET or CT [77]. The phase III PATINA trial wants to investigate the advantage of combining palbociclib with trastuzumab with or without pertuzumab and ET maintenance therapy after induction therapy in the first-line setting for HR-positive/HER2-positive metastatic BC [78].

There are different ongoing clinical trials concerning HR-positive/HER-positive metastatic BC testing new molecules (such as pyrotinib, a new TKI against HER2, or dalpiciclib, a new CDK4/6I) and combinations, and they are summarized in Table 1.

**Table 1:** Ongoing clinical trials in HR-positive/HER-positive metastatic BC. Abbreviations: PFS, progression-free survival; ORR, objective response rate; CBR, clinical benefit rate; DOR, duration of response; OS, overall survival; AEs, adverse events, and safety; QoL, quality of life; DCR, disease control rate; PROs, patient-reported outcomes; TTR, time to response; CNS, central nervous system; G, grade; PK, pharmacokinetic; CT, chemotherapy; ET, endocrine therapy.

Trial	Study Phase	Treatment Line	Study Drugs	End-Points	Ref.
NCT03913234	Phase Ib/II	First	Ribociclib+ Letrozole+ Trastuzumab	Primary: PFS Secondary: ORR, OS, QoL	[79]
YOUNGBC-22 (NCT05574881)	Phase II	First	Dalpiciclib+ Fulvestrant+ Trastuzumab+ Pertuzumab	Primary: PFS Secondary: ORR, CBR, DOR, OS, AEs	[80]
BREAST-Pyrotinib (NCT03910712)	Phase II	First	Aromatase inhibitor+ Trastuzumab +/− Pyrotinib	Primary: PFS Secondary: ORR, DOR, OS, TTR, CBR, QoL, AEs	[81]
NCT04088110	Phase II	First	Trastuzumab+ Aromatase inhibitors+ Pyrotinib	Primary: PFS Secondary: ORR, DOR, OS	[82]
FAVOR trial (NCT04337658)	Phase III	First	Trastuzumab+ Pertuzumab + Fulvestrant vs. Capecitabina	Primary: PFS Secondary: CBR, OS, AEs	[83]
DETECT V/ CHEVENDO trial (NCT02344472)	Phase III	First	Trastuzumab+ Pertuzumab+ + CT vs. Ribociclib + ET	Primary: AEs Secondary: OS, PFS, ORR, incidence of CNS metastases, QoL, DCR	[77]
PATINA trial (NCT02947685)	Phase III	First line after induction therapy	Trastuzumab+ Pertuzuman+ ET +/− Palbociclib	Primary: PFS Secondary: ORR, DOR, OS, CBR, PROs, AEs, incidence of CNS metastases	[78,84]
NCT04034589	Phase II	First line and beyond	Pyrotinib+ Fulvestrant	Primary: PFS Secondary: ORR, CBR, OS, AEs, QoL	[85]
NCT03054363	Phase Ib/II	Second and beyond	Tucatinib+ Letrozole+ Palbociclib	Primary: PFS, AEs Secondary: PK	[86]
NCT04646759	Phase III	Second and beyond	Pyrotinib + Fulvestrant vs. Capecitabine	Primary: PFS, incidence of G3 hand-foot syndrome Secondary: ORR, OS, CBR, QoL, Safety	[87]
MonarcHER (NCT02675231)	Phase III	Third line and beyond	Abemaciclib+ Trastuzumab vs. Abemaciclib+ Trastuzumab+ Fulvestrant vs. Trastuzumab+ SoC CT	Primary: PFS Secondary: ORR, DOR, OS, DCR, CBR, QoL	[76,88]

While the combination of anti-HER2 antibodies and ET has been extensively researched (as shown in Table 1), there is a lack of data regarding the use of ADCs combined with ET. However, in clinical practice, anti-HER2 treatments (including both antibodies and ADCs) are frequently administered together, particularly in the metastatic setting for patients with HR-positive/HER2-positive BC. This practice is especially prevalent after observing a clinical response and as part of maintenance therapeutic strategies.

## 5. A New Entity: HER2low BC

### 5.1. Biology of HER2low BC

HER2 expression can be assessed by IHC and ISH. Based on current guidelines, HER2-low BC is defined as BC with low levels of HER2 and is identified by IHC score 1+ or 2+ with negative ISH, defined as ISH < 2.0 or HER2 gene copy number < 4 [3]. Score 2+ is defined by a weak or moderate complete membrane staining in >10% of tumor cells or an intense membrane staining in ≤10% of tumor cells; score 1+ is represented by faint or barely perceptible incomplete membrane staining in >10% of tumor cells [89].

HER2-low BC includes heterogeneous tumors, such as HR-positive BCs as well as triple-negative BCs (TNBCs). Around 30–60% of the BCs traditionally defined as ‘HER2-negatives’ are HER2-low tumors [90]; particularly, around 40–65% of HR-positive and 23–40% TN BCs are considered HER2-low [91,92,93]. A total of 68% of HER2-low tumors are HER2 IHC 1+, regardless of HR status [90].

HER2-low expression seems to increase with ER expression and appears to be enriched in the advanced setting [91]; indeed, HER2 expression is dynamic and can change during disease progression with up to 40% discordance between primary and metastatic tumors [94,95].

The defining of clinicopathologic and prognostic characteristics of HER2-low subtype have led to conflicting results and are still inconclusive [90,95,96,97]. An important clinical question is if the HER2-low BC represents a distinct biological entity, different from HER2-0 tumors, and if this subgroup has a specific prognostic profile [98].

A large retrospective study analyzing the PAM50 data of 3689 BC patients evidenced that HR-positive/HER2-low tumors are characterized by a high expression of luminal-related genes, while basal-like genes and proliferation-related genes were downregulated in HER2-low tumors compared to HER2-0 tumors. On the other hand, no significant subtype distribution was observed in TNBCs, suggesting that HR-positive HER2-low tumors are more distinct biological entities, in contrast to HER2-low TNBC tumors [90]. Other analyses confirmed an association between HR-positive/HER2-low and luminal intrinsic subtype [99,100].

Multiple studies have been conducted to assess the prognostic implications of HER2-low expression in early and advanced BC. As compared to HER2 IHC 0/1+ BCs, HER2 IHC 2+/ISH-negative BCs are significantly associated with a larger tumor size, positive lymph node status, higher histological grade, and an increased Ki-67 index; compared with HER2 IHC 0 tumors, the HER2-low BCs exhibit a lower histological grade [90,97].

A pooled analysis involving 2310 HER2-negative BC patients from four prospective neoadjuvant clinical trials evidenced that HER2-low status seems to have a prognostic role; particularly, low expression of HER2 was significantly associated with a better DFS and OS, a lower number of grade III tumors, lower Ki-67 scores, and a reduced number of TP53 mutations compared with HER2 IHC score 0 BCs [100]. In addition, pCR rates were lower in HER2-low tumors than in HER2-0, especially in the HR-positive subgroup. However, HER2-low BCs have shown significantly longer survival compared to HER2-0 BC, and this evidence was seen in the HR-negative subgroup, while in the HR-positive subgroup, no survival benefit was observed [100].

Different studies tried to analyze the association between HER2-low expression and survival in HR-positive and HR-negative BC, and the results are controversial.

The Asian Breast Cancer Cooperative Group found a better prognosis for HER2-low BC compared with HER2 IHC score 0 in the early setting, although there was a modest absolute difference, mainly driven by HER2 IHC score 1+ tumors. No significant difference in RFS and OS was described between HER2 IHC score 2+/ISH-negative and HER2 IHC score 0 [101]. In the advanced setting, a better OS has been evidenced for HER2-low BC in comparison with HER2-0 BC in a cohort of 15.054 patients with HER2-negative BC, and the findings were seen in the HR-negative subgroup, although smaller cohorts have not demonstrated a significant difference [90,101,102]. Gampenrieder et al. studied 1729 patients with metastatic BC and highlighted that low HER2 expression did not significantly influence OS in the HR-positive or triple-negative subgroups [92]. In an Italian cohort, 5-year overall survival (OS) for HER2 2+/FISH- BCs was lower than that for HER2 IHC 0 or 1+ tumors (*p* = 0.03). In addition, HER2 2+/ISH-negative BCs have more likely late-stage diagnosis (*p* = 0.04), high grade (*p* < 0.0001), or high Ki67 (*p* < 0.0001) [103].

The level of expression of ER seems to impact the prognosis of HER2-low BC. A significant positive association between ER expression and HER2-low expression was found in a cohort of 5235 patients with early HER2-low BC at the Dana-Farber Brigham Center; HER2-low expression did not seem to play a distinct prognostic role, and the authors found no statistically significant difference in the pCR rate and in OS between HER2-low and HER2-0 tumors, regardless of HR+ status [91,101,102]. An Italian Cancer Registry study evaluated 3633 HER2low BC and divided ER status into low (1–9%), moderate (10–79%), and high (80–100%). ER-low/moderate tumors were associated with poorer OS in comparison with ER-high BCs (*p* < 0.0001). ER-high tumors with HER2 2+/FISH- status had a worse OS (*p* = 0.04), while this association was not found among ER-low/moderate BCs (*p* = 0.21) [103,104].

### 5.2. Therapeutic Options for HER2low BC

Previously, HER2-low BCs were treated as HER2-negative tumors.

The first treatment directed toward HER2 studied for the HER2-low BC was trastuzumab. The phase III NSABP B-47 trial randomized 3270 patients with invasive, high-risk, HER2-low BC to adjuvant CT with or without trastuzumab for one year. At a median follow-up of 46 months, no difference was observed in terms of iDFS between the groups (hazard ratio 0.98, 95% CI: 0.76–1.25, *p* = 0.85), irrespective of HR status, HER2 IHC expression, or lymph node involvement [105]. Similarly, pertuzumab and T-DM1 treatment showed a weak activity in HER2low BC [98]. Based on this evidence, the indication for HER2-directed therapies remained limited to HER2-positive disease, according to the 2018 ASCO/CAP guidelines [106].

However, the development of more potent HER2-directed therapies such as novel antibody-drug conjugates (ADCs) has reshaped the treatment paradigm of HER2-low BC.

A phase Ib study of 54 patients (NCT02564900) explored the efficacy and safety of T-DXd) in patients with advanced HER2-low BC, with an ORR of 37% (95% CI, 24.3–51.3%), a median duration of response (DOR) of 10.4 months, a median PFS of 11.1 months, and a median OS of 29.4 months [107]. Phase III DESTINY-Breast04 trial revolutionized the landscape of treatment of HER2-low BC, demonstrating the efficacy of T-DXd in pre-treated metastatic HER2-low patients. T-DXd significantly improved PFS (9.9 vs. 5.1 months, HR 0.50, 95% CI: 0.40–0.63, *p* < 0.001) and OS (23.4 vs. 16.8 months, hazard ratio 0.64, 95% CI 0.49–0.84, *p* = 0.001) in patients with HER2-low BC, regardless of the HR status [108]. This trial included 557 pre-treated patients randomly assigned to receive T-DXd or standard CT. Benefit from T-DXd was observed in HR-positive patients (N = 494, 88.7% of patients) with a median PFS of 10.1 vs. 5.4 months (hazard ratio 0.51, *p* < 0.001) and a median OS of 23.9 vs. 17.5 months (hazard ratio 0.64, *p* = 0.003). An exploratory analysis confirmed the benefit also in patients with TNBC (N = 58, 11.3% of patients), with a median PFS of 8.5 vs. 2.9 months (hazard ratio 0.46) and a median OS of 18.2 vs. 8.3 months (hazard ratio 0.48) with T-DXd compared with CT. The results were observed irrespective of HER2 expression (IHC 1+ or 2+), although a slightly longer PFS (10.3 vs. 10.1 months) and a higher reduction in the risk of progression (52% vs. 45%) were noted in patients with HER2 IHC score 1+ in comparison with the 2+ subgroup [108]. The DESTINY-Breast04 trial led to the approval of T-DXd by the USA Food and Drug Administration (FDA) and by EMA (European Medicines Agency) for patients with pretreated HER2-low metastatic BC who have received at least one prior line of CT [109,110]. The ongoing DESTINY-Breast06 will explore the role of T-DXd in HR-positive patients after at least 2 previous lines of ET for the metastatic setting and previous treatment with CDK4/6Is [111].

Sacituzumab govitecan (IMMU-132) is another treatment option for HR-positive HER2-low metastatic BC, based on the positive results of the TROPICS-02 clinical trial, that showed a PFS (6.4 vs. 4.2 months, HR 0.58, *p* < 0.01) benefit for sacituzumab govitecan over the CT of physician’s choice (TPC) [112]. About 52% of enrolled patients have HR-positive HER2-low BC and half received sacituzumab govitecan. ORR was 26% versus 12% with sacituzumab govitecan versus TPC, respectively. In the HER2 IHC 0 group, ORR was 16% versus 15%, respectively. Median PFS for the IHC1+ and IHC2+ subgroups was 7 versus 4.3 months (hazard ratio 0.57) and 5.6 versus 4 months (hazard ratio 0.58), respectively [113]. The ASCENT trial confirmed the efficacy of sacituzumab govitecan in HER2-low metastatic BC, showing a significantly longer PFS (6.2 vs. 2.9 months, hazard ratio 0.44, 95% CI: 0.27–0.72, *p* = 0.002) and OS (14 vs. 8.7 months, hazard ratio 0.43, 95% CI: 0.28–0.67, *p* < 0.001) with sacituzumab govitecan over the CT of physician’s choice [114].

Other new ADCs have also shown clinical activity in pretreated HER2-low metastatic BC patients.

Trastuzumab duocarmazine (SYD985) was tested in a phase I trial enrolling 49 patients with HER2-low BC (32 were HR-positive and 17 had TNBC). In the two cohorts, an ORR was achieved in 28% of patients with HR-positive BC and in 40% of TNBC patients. The median PFS was 4.1 in the HR-positive cohort and 4.9 months in TNBC cohort, respectively [115].

Disitamab vedotin (RC48) showed activity in HER2-low metastatic BC patients [116]. A pooled analysis of two phase I studies (NCT02881138 and NCT03052634) evaluated the efficacy and safety of RC48 in HER2-positive and HER2-low BC. The median PFS was 5.7 months and the ORR was 39.6% in HER2-low BC, of which there were 42.9% in patients with HER2 IHC 2+/FISH- and 30.8% in HER2 IHC 1+ patients [117]. A phase III trial (NCT04400695) exploring the efficacy and safety of RC48 in HER2-low BC is ongoing [118].

MRG002 has been investigated in a phase II trial including pretreated HER2-low metastatic BC patients. HER2 IHC 1+ and HER2 IHC 2+/ISH-negative BC patients obtained similar ORRs, disease control rates, and mPFSs (33%, 75% and 5.6 months, respectively) [119].

Ongoing trials are evaluating the activity of ADCs combined with immunotherapy. The phase 1b/2 BEGONIA trial, investigating the combination of Durvalumab and T-DXd in HER2-low TNBC patients, shows promising early safety and efficacy results [120]. The NCT04042701 trial exploring the combination of T-DXd with pembrolizumab is ongoing [121].

Additional treatments for HER2-low BC are emerging and are summarized in Table 2.

## 6. Conclusions

In conclusion, HR-positive/HER2-positive BC requires multi-agent treatments that aim to block HER2 and ER pathways simultaneously.

In the early setting, neoadjuvant CT with antiHER2 blockage is the standard of care. Trastuzumab-based treatments are also used with or without CT and/or pertuzumab in the adjuvant setting. ET is typically administered in adjuvant setting and it is prescribed based on the same indications used for high-risk BC patients. In premenopausal patients, extended ET for 10 years with tamoxifen with or without OFS is the most consolidated strategy; less evidence is available for AI + OFS options. In postmenopausal patients, the adjuvant treatment requires AI for a duration of 5 to 7 years (Figure 2).

In advanced settings, there is no evidence of better ET in combination with anti-HER2 agents, but the combination treatment seems to improve the outcome in patients with HR-positive/HER2-positive metastatic BC.

Prospective trials are evaluating the efficacy of anti-HER2 treatment in combination with new endocrine agents to block the pathways involved in this pathology in the early setting to avoid/reduce the use of CT in the early as well as metastatic settings. The most promising evidence emerges from clinical trials combining trastuzumab and CDK4/6I. Additionally, other combinations involving TKIs are also being examined in ongoing clinical trials.

In the end, combination treatments with ET and anti-HER2 agents seem to have an essential role in the management of HER2-low metastatic BC patients as well. Ongoing clinical trials aim to further solidify this evidence.

## Figures and Tables

**Table 2:** New drugs in ongoing trials in HER2low BC. Abbreviations: Ig: immunoglobulin, mAb: monoclonal antibody; ADC: antibody-drug conjugate.

Drug	Clinical Trial	Phase Trial	Action and Rational	Ref
GP2 peptide		Preclinical	HER2-derived peptide vaccine	[122]
ZW49		Preclinical	Biparatopic ADC, which combines ZW25 with linker-drug conjugated via disulfides containing a cleavable linker and novel auristatin payload	[123]
Zanidatamab (ZW25)	NCT02892123	Phase I	Humanized, bispecific, Ig G isotype 1-like, mAb directed against the juxtamembrane extracellular domain and the dimerization domain of HER2.	[124,125]
ARX788	ACE-PanTumor-01	Phase I/II	ADC with anti-HER2 mAb and a potent tubulin inhibitor payload AS269	[126,127]
A166	CTR20181301	Phase I	ADC composed of a cytotoxic drug (Duostatin-5, anti-microtubule agent) with site-specific conjugation to trastuzumab via a stable protease-cleavable valine citrulline linker	[128,129]
PF-06804103	NCT03284723	Phase I	Anti-HER2 ADC with auristatin payload	[130]
FS-1502	NCT03944499	Phase I	ADC against HER2 with a cleavable β-glucuronide linker and an antimitotic agent (monomethyl auristatin F)	[131]
AE37 + GM-CSF	NCT00524277	Phase I	Peptide vaccine	[132]
ARX788	ACE-PanTumor-01	Phase I/II	ADC with anti-HER2 mAb and a potent tubulin inhibitor payload AS269	[126,127]

## Data Availability

Not applicable.
